# Cardiovascular and Thromboembolic Risk of Janus Kinase Inhibitors Compared to Other Disease-Modifying Drugs in Patients with Rheumatoid Arthritis: A Systematic Review and Meta-Analysis

**DOI:** 10.3390/jpm16020113

**Published:** 2026-02-13

**Authors:** Diomidis C. Ioannidis, Efthymia Maria Kapasouri, Vassilios S. Vassiliou, Eleana Ntatsaki

**Affiliations:** 1 Department of Medicine, Ipswich Hospital, East Suffolk and North Essex Foundation Trust, Heath Road, Ipswich IP4 5PD, UK; 2 Norwich Medical School, University of East Anglia, Rosalind Franklin Road, Norwich NR4 7UQ, UK; 3 Department of Cardiology, Norfolk and Norwich University Hospital, Colney Lane, Norwich NR4 7UY, UK; 4 Department of Rheumatology, Ipswich Hospital, East Suffolk and North Essex Foundation Trust, Heath Road, Ipswich IP4 5PD, UK

**Keywords:** rheumatoid arthritis, cardiovascular event, Janus Kinase inhibitors, MACE, VTE

## Abstract

**Background/Objectives**: Janus Kinase inhibitors (JAKi) are an effective treatment option for rheumatoid arthritis (RA); however, emerging concerns regarding cardiovascular and thromboembolic risk have prompted further investigation. We conducted a systematic review and meta-analysis to compare the risk of major adverse cardiovascular events (MACE) and venous thromboembolism (VTE) in patients receiving JAKi versus other disease-modifying anti-rheumatic drugs (DMARDs). **Methods:** Following PRISMA 2020 guidelines and a preregistered protocol, we systematically searched PubMed, Embase, and the Cochrane Library. Observational studies and randomized controlled trials (RCTs) reporting MACE or VTE among adults with RA treated with JAKi or comparator DMARDs were included. Hazard ratios (HRs) from observational studies and odds ratios (ORs) from RCTs were pooled using fixed- or random-effects models depending on heterogeneity. A sensitivity analysis was conducted for participants aged ≥ 65 years. **Results:** Twenty-five observational studies and eight RCTs were included. Across observational studies, the pooled HRs for MACE showed no significant difference between JAKi and other DMARDs, HR = 0.98, 95% CI = 0.85–1.13. This finding remained consistent in individuals aged ≥ 65 years. No increase in MACE risk was observed across RCTs, OR = 1.27, 95% CI = 0.89–1.81. In contrast, JAKi use was associated with a significantly higher risk of VTE in the observational studies (HR = 1.32, 95% CI = 1.08–1.61) but not in the RCTs (OR = 1.69, 95% CI = 0.94–3.02). **Conclusions:** JAKi use does not appear to increase the risk of MACE compared to DMARDs, including in older adults, but may be associated with a higher risk of VTE. These findings highlight the importance of a personalized approach when considering JAKi therapy, incorporating structured cardiovascular and thrombotic risk assessment, patient preferences, and mitigation of modifiable risk factors.

## 1. Introduction

Rheumatoid arthritis (RA) is a chronic, systemic inflammatory disorder primarily affecting the small synovial joints, although any joint may be involved. The condition affects approximately 1–2% of the global population and, if inadequately controlled, may lead to progressive joint destruction that may necessitate surgical intervention, contribute to work disability, and result in a substantial reduction in overall quality of life [1].

Early and effective treatment of rheumatoid arthritis is therefore essential to mitigate disease progression and reduce the risk of systemic complications. Conventional synthetic disease-modifying anti-rheumatic drugs (csDMARDs), including methotrexate, sulfasalazine, hydroxychloroquine, and leflunomide, remain the cornerstone of therapy. Biologic DMARDs (bDMARDs), such as Tumor Necrosis Factor inhibitors, B-cell–depleting agents, and interleukin inhibitors, are used in patients with inadequate responses to conventional therapy [2].

**Figure 1:**
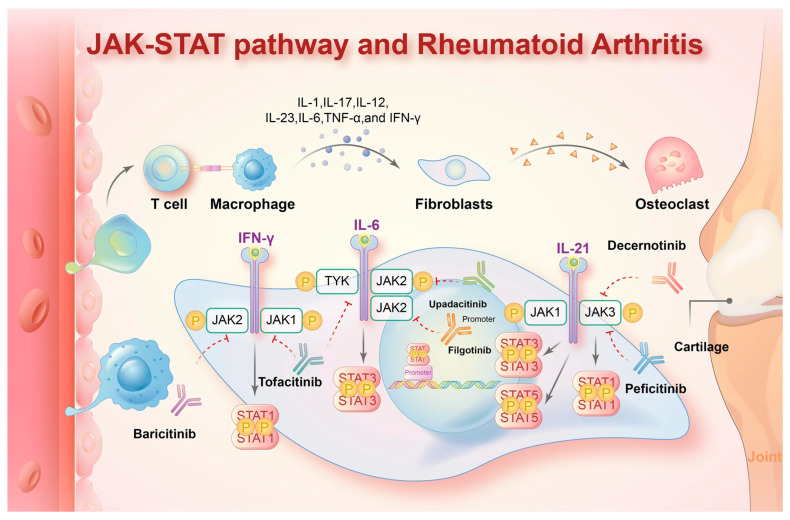
JAK-STAT pathway and receptors for the mechanistic action of JAKi. Reproduced from Xue et al. [16] under the Creative Commons Attribution 4.0 International License.

Despite the availability of multiple therapeutic options, a subset of patients with RA continue to experience active disease and persistent symptoms regardless of treatment with several disease-modifying agents. Therefore, ongoing research continues to focus on identifying additional therapeutic strategies for patients with rheumatoid arthritis. Janus Kinase inhibitors (JAKi) are targeted synthetic DMARDs and represent a relatively novel class of anti-rheumatic agents introduced into clinical practice [3]. These agents target the JAK–STAT signaling pathway, a key mediator of inflammatory responses, as shown in Figure 1. Activation of JAK1 by interleukin 6 promotes synoviocyte proliferation and the downstream cascade leading to joint inflammation and destruction. JAK2 is involved in hematopoietic signaling through cytokines, such as erythropoietin and granulocyte colony-stimulating factor, whereas JAK3 plays a central role in innate lymphoid cell–mediated immune responses [4].

The first JAKi, tofacitinib, for RA was approved by the US Food and Drug Administration (FDA) in 2012 [5]. Subsequently, JAKi were also approved by the UK National Institute for Health and Care Excellence (NICE) [6] and the European Medicines Agency (EMA) [7]. JAKi are generally reserved for patients with moderate to severe rheumatoid arthritis and who had inadequate response to csDMARDs and bDMARDs. In Table 1, we summarize the existing recommendations of the FDA, NICE, and EMA for the approved JAKi [5,6,7,8,9,10,11,12,13,14,15].

Whilst JAKi have demonstrated significant efficacy in randomized clinical trials (RCTs), emerging safety data have raised concerns regarding an increased risk of major adverse cardiovascular events (MACE) and venous thromboembolism (VTE), particularly among patients older than 65 years, especially amongst those with preexisting cardiovascular conditions (e.g., myocardial infarction, heart failure, or stroke) and those with elevated cardiovascular risk, such as patients with hypertension, dyslipidaemia, physical inactivity, and even current or former smokers [4].

**Table 1:** Dates of approval and specific indications for use of each JAKi. FDA = US Food and Drug Administration; NICE = UK National Institute for Health and Care Excellence; and EMA = European Medicines Agency.

JAKi	FDA	NICE	EMA
Tofacitinib	Approved in 2012. Licensed for adult patients with moderately to severely active RA who have had an inadequate response or intolerance to one or more TNFi.	Approved in 2017. Licensed for adults with severe disease who responded inadequately to two csDMARDs or one bDMARD and cannot have rituximab.	Approved in 2018. Licensed for adults with moderate to severe disease who responded inadequately to one bDMARD.
Baricitinib	Approved in 2018. Licensed for adults with moderately to severely active rheumatoid arthritis who have had an inadequate response to one or more TNF blockers.	Approved in 2017. Licensed for adults with severe disease who responded inadequately to two csDMARDs or one bDMARD and cannot have rituximab.	Approved in 2017. Licensed for adults with moderate to severe rheumatoid arthritis who did not respond to DMARDs or if the patient cannot take these medicines.
Upadacitinib	Approved in 2020. Licensed for adults with moderately to severely active rheumatoid arthritis who have had an inadequate response or intolerance to one or more TNF blockers.	Approved in 2020. Licensed for adults with severe disease who responded inadequately to two csDMARDs or one bDMARD and cannot have/or had inadequate response to rituximab.	Approved in 2019. Licensed for adults with moderate to severe rheumatoid arthritis who did not respond to DMARDs or if the patient cannot take these medicines.
Filgotinib	Not yet approved.	Approved in 2021. Licensed for adults with moderate to severe disease who responded inadequately to two csDMARDs or severe disease with inadequate response to one bDMARD and cannot have/or had inadequate response to rituximab.	Approved in 2020. Licensed for adults with moderate to severe RA.

The objective of this systematic review and meta-analysis was to synthesize the available evidence regarding adverse cardiovascular outcomes among patients treated with JAKi, a population of individuals with RA who already demonstrate an elevated baseline cardiovascular risk. By quantifying the magnitude of this risk across contemporary studies, our aim was to provide a clearer understanding of the extent to which JAKi therapy may contribute to cardiovascular events within this vulnerable group. Recognition of such risks in routine clinical practice is essential, as it would not only prompt clinicians to adopt a more vigilant approach to monitoring, but it would also support the integration of personalized cardiovascular risk appraisals into therapeutic discussions. Given that patients with RA represent a cohort with a substantial and heterogeneous cardiovascular burden driven by chronic inflammation, traditional risk factors, and treatment-related influences, a more nuanced, patient-centered assessment is warranted. Ultimately, the identification of individuals at heightened risk may enable clinicians to offer tailored cardiovascular risk-reduction strategies, thereby aligning treatment decisions with the principles of precision medicine and optimizing long-term outcomes. For the purposes of this review, we will refer to JAKi as an entity on its own, and when using the term DMARD, this covers non-JAKi csDMARD and bDMARD categories.

## 2. Materials and Methods

This systematic review was conducted in accordance with the Preferred Reporting Items for Systematic Reviews and Meta-Analyses (PRISMA) 2020 guidelines [17]. The review protocol was registered with the Open Science Framework (Registration Number: Q7ZWG) [18]. A comprehensive literature search was performed in three electronic databases: PubMed, Embase (via Ovid), and the Cochrane Library, from inception to 24 July 2025. The search strategy included combinations of the following terms:

(Arthritis, Rheumatoid [Mesh] OR rheumatoid arthritis OR RA) AND (Janus Kinase Inhibitors [Mesh] OR JAK inhibitors OR tofacitinib OR baricitinib OR upadacitinib OR filgotinib OR peficitinib) AND (cardiovascular [Mesh] OR Heart [Mesh] OR Cardiac [Mesh]).

In Embase, the search was limited to full-text articles and human studies, excluding article reprints. No additional restrictions were applied. The complete search strategy is provided in Supplementary Table S1. Studies were included if they met the following criteria:

(1) Observational studies (case–control, prospective, or retrospective cohort) or RCTs.
(2) Human studies published in English.
(3) Studies involving adults (>18 years old) diagnosed with RA.
(4) Studies including participants who were treated with JAKi in one group and participants treated with csDMARDs, TNF inhibitors, or other bDMARDs in a different group.
(5) Studies reporting major adverse cardiovascular events (MACE) and/or venous thromboembolism (VTE) as outcomes. If a study reported either MACE or VTE but not both, it was still included.

Conference abstracts, case reports, and letters to the editor without original data were excluded. Studies were also excluded if JAKi were investigated for indications other than RA (e.g., inflammatory bowel disease or atopic dermatitis), or if MACE or VTE outcomes were not included.

Full references and abstracts identified through the database searches were imported using Covidence, a dedicated systematic review software. Duplicate records were automatically removed by the software. After deduplication, two reviewers (D.I. and V.V.) independently screened titles and abstracts according to the predefined selection criteria to determine their eligibility for full-text review. Disagreements were adjudicated by E.N.

From each included study, the following data were extracted: first author, publication date, and type of study (observational or RCT). Demographic data included the number of participants receiving JAKi or comparator treatments, mean age, proportion of female participants, proportion of smokers, prevalence of diabetes, and history of prior stroke or myocardial infarction. Outcome data included the incidence of overall MACE, myocardial infarction, stroke, cardiovascular death, and venous thromboembolism (DVT or PE). As there was variability among studies in the reporting format of results (e.g., hazard ratio, risk ratio, incidence rate, incidence ratio, or absolute number of events) the results were recorded, and the format documented. For the observational studies, the results from a multivariable regression model or propensity-matched analysis were used preferentially if available to allow us to investigate the independent effect of JAKi. The risk of bias for observational studies was assessed using the Joanna Briggs Institute Critical Appraisal Checklist (JBI) [19] and for RCTs with Cochrane Risk of Bias (RoB) tool 2 [20], as shown in Supplementary Figures S1 and S2, respectively. Each item in the appraisal tools was rated as “Yes,” “No,” or “Unclear.” The study quality was independently assessed by two reviewers. Furthermore, we *a priori* planned to use a fixed-effects model if heterogeneity was I^2^ < 50% and a random-effects model if heterogeneity was I^2^ ≥ 50%.

## 3. Results

**Figure 2:**
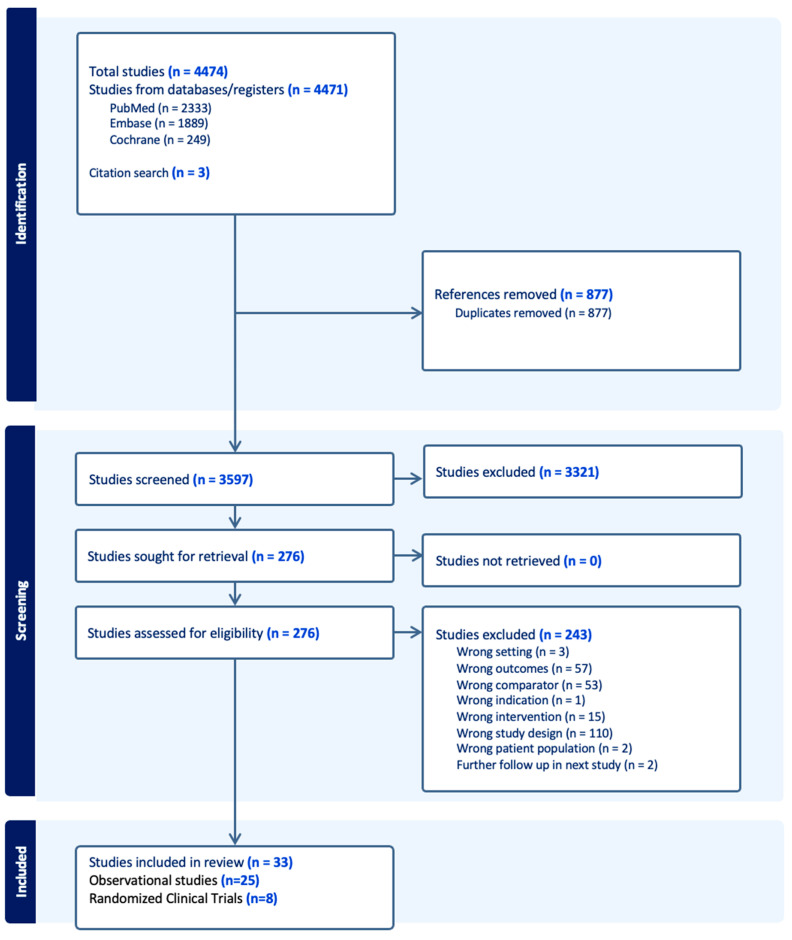
PRISMA flow chart.

The full selection process is summarized in the PRISMA flow chart (Figure 2) and PRISMA checklist in Supplementary Table S2.

### 3.1. Risk of Bias Observational Studies

A total of 25 observational studies were evaluated for their risk of bias using the JBI Critical Appraisal Checklist *(*Supplementary Figure S1) [21,22,23,24,25,26,27,28,29,30,31,32,33,34,35,36,37,38,39,40,41,42,43,44,45]. Overall, the risk of bias was low, with most studies meeting all 11 criteria on the checklist. Only two studies did not identify or address potential confounding factors [23,25].

#### 3.1.1. Risk of MACE Overall Population

A total of 22 studies presented outcomes related to MACE in their results [21,22,23,24,25,26,28,29,30,31,32,33,34,35,36,38,39,40,41,43,44,45]. Demographics are summarized in Supplementary Table S3 of the Supplementary Materials with the results in Supplementary Table S4. Sample sizes varied between studies. Hirose et al. was the smallest study with a total of 370 participants, with 183 in the abatacept group and 187 in the tofacitinib group [30]. Kwosrow-Khavard had the largest sample comprising 102,263 patients, most of which were in the TNFi group [32]. Min et al. had two set of populations: firstly, in Set 1 they compared JAKi vs. bDMARDs in patients who had never received JAKi or bDMARDs before, and secondly, in Set 2 they compared JAKi vs. bDMARDs in patients who were on bDMARDs previously [35]. Aymon et al. had the highest JAKi subgroup with a total of 16,417 participants [21]. Song et al. had the youngest mean participant age in both intervention and control groups, with 48.1 years old in the JAKi group and 48.5 years old in the bDMARD group [41]. For the rest of the studies, the mean age exceeded 50 years old in both participant and intervention groups. The proportion of female patients was significantly higher in all studies, secondary to the prevalence of RA being significantly increased in females. Specifically, Deakin et al. had the lowest female percentage of 68% in the adalimumab group [26]. In the rest of the studies, the female proportion was greater than 70% in both control and comparator groups. A total of 17 studies compared JAKi directly with Tumor Necrosis Factor inhibitors (TNFi), three studies compared JAKi to TNFi and JAKi to the rest of bDMARDs separately [39,41,43]. Moreover, two studies compared JAKi to bDMARDs in general [23,33], and another study compared JAKi to abatacept [30]. Overall, the risk of MACE was comparable between JAKi and bDMARD, and this was the case for most of the trials. Notably, Bower et al. and Min et al. Set 1 population, -i.e., JAKi vs. bDMARDs in patients who had never received JAKi or bDMARDs before-, reported statistically significant lower risk of MACE in the JAKi group [22,35]. Sakai et al. demonstrated statistically significant higher risk of MACE in the JAKi without methotrexate (MTX) vs. TNFi without MTX groups, and in the JAKi with MTX vs. TNFi with MTX groups as well as in the JAKI without MTX vs. non TNFI without MTX groups [39].

**Figure 3:**
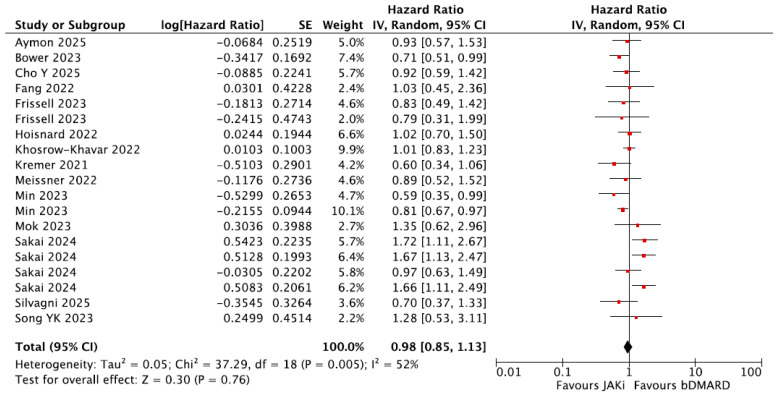
Forest plot for risk of MACE from observational studies. For the Fang et al. study, in the absence of MACE data, coronary heart disease events were included in the meta-analysis: bDMARDs vs. JAKi. bDMARDs = Biologic Disease-Modifying Anti-Rheumatic Drugs; JAKi = Janus Kinase inhibitors [21,22,24,28,29,31,32,33,34,35,36,39,40,41].

Of these, 14 studies provided HRs comparing the risk of MACE between patients receiving JAKi and a reference group of patients receiving bDMARDs, and these were included in the meta-analysis [21,22,24,28,29,31,32,33,34,35,36,39,40,41]. Both populations from the Min et al. study mentioned above were incorporated in the meta-analysis. Sakai et al. compared JAKi vs. TNFi monotherapy; JAKi with MTX vs. bDMARDs with MTX; JAKi vs. non-TNFi bDMARD monotherapy; and JAKi with MTX vs. non-TNFi bDMARDs with MTX [39]. All four of these comparisons were incorporated in the meta-analysis (Figure 3). Frisell et al. compared etanercept vs. baricitinib and etanercept vs. tofacitinib. Both comparisons were included in this meta-analysis [29].

Ultimately, incorporating 19 distinct populations, the meta-analysis showed no difference in the risk of MACE between the JAKi and bDMARD groups with overall HR = 0.98, 95% CI = 0.85–1.13.

#### 3.1.2. Risk of MACE over 65 Years of Age

**Figure 4:**
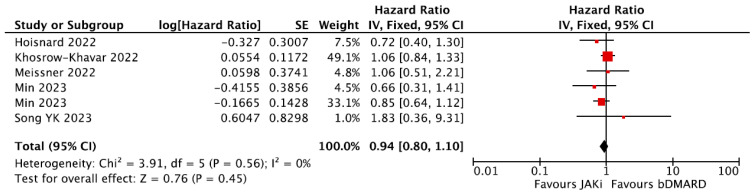
Forest plot for risk of MACE in patients over 65 years old from observational studies. bDMARDs = Biologic Disease-Modifying Anti-Rheumatic Drugs; JAKi = Janus Kinase inhibitors [31,32,34,35,41].

Given the concerns about higher risks in patients over the age of 65, a pre-specified sensitivity analysis was undertaken including populations comprising only patients over the age of 65 years old. Five studies reported HRs for the risk of MACE among participants aged 65 years or older receiving JAKi compared with bDMARD, which was the common reference used across all studies [31,32,34,35,41]. A sensitivity meta-analysis was undertaken (Figure 4) showing no difference in the risk of MACE between the bDMARDand JAKi groups with a pooled HR = 0.94, 95% CI = 0.80–1.10. Similarly to the overall population, the Min et al. study had two distinct populations (one considering JAKi-naïve individuals and one including patients who had received JAKi or bDMARD previously), both of which were included in this sensitivity meta-analysis.

#### 3.1.3. Risk of MACE in TNFi vs. Baricitinib and TNFi vs. Tofacitinib

**Figure 5:**
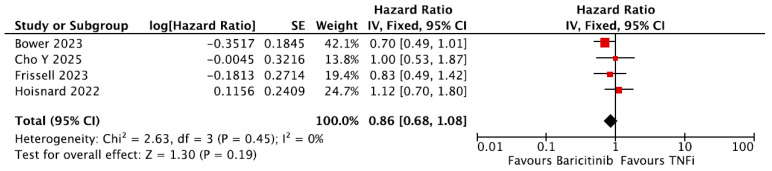
Forest plot for risk of MACE from observational studies: TNFi vs. baricitinib. TNFi = Tumor Necrosis Factor inhibitors [22,24,29,31].

A total of four studies conducted subgroup analyses of the risk of developing MACE in a baricitinib group versus a TNFi group [22,25,29,31]. A meta-analysis was performed and showed no difference in the risk of developing MACE between patients receiving baricitinib and those receiving TNFi (pooled HR = 0.86, 95% CI = 0.68–1.08, Figure 5).

**Figure 6:**
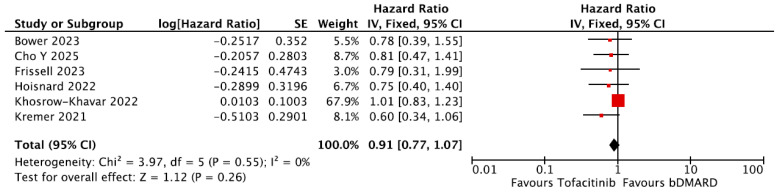
Forest plot for risk of MACE from observational studies: bDMARDvs. tofacitinib. bDMARD= Biologic Disease-Modifying Anti-Rheumatic Drugs [22,24,29,31,32,33].

A total of six studies conducted subgroup analyses comparing the risk of developing MACE in patients treated with tofacitinib versus those treated with a BDMARD [22,25,29,31,32,33]. A meta-analysis was performed and showed no difference in the risk of developing MACE between the tofacitinib and BDMARD groups (pooled HR = 0.91, 95% CI = 0.77–1.07, Figure 6) [29].

#### 3.1.4. Risk of VTE Overall Population

**Figure 7:**
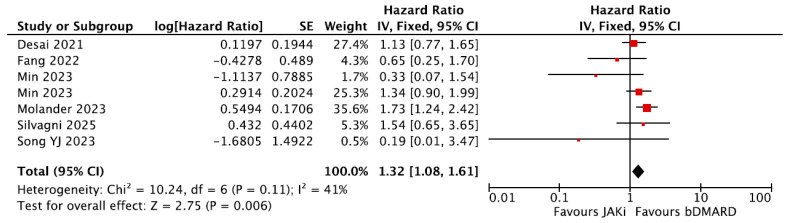
Forest plot for risk of VTE from observational studies: bDMARDs vs. JAKi. bDMARDs = Biologic Disease-Modifying Anti-Rheumatic Drugs; JAKi = Janus Kinase inhibitors [27,28,35,37,40,42].

Six studies presented the HRs of VTE between a reference group of people receiving bDMARDs and a group of people receiving JAKi [27,28,35,37,40,42]. A meta-analysis was performed and showed a statistically significant increased risk of VTE in the JAKi group (pooled HR = 1.32, 95% CI = 1.08–1.61, Figure 7). Both populations from Min et al. as described above were eligible and included in the meta-analysis.

### 3.2. Results of Randomized Clinical Trials

#### 3.2.1. Risk of Bias of Randomized Clinical Trials

Eight RCTs met the inclusion criteria and were included in this manuscript. They were assessed for bias using the Cochrane ROB2 tool (Supplementary Figure S2) [46,47,48,49,50,51,52,53]. The overall risk of bias was low, with most of the trials meeting all seven criteria of the tool. One trial was not blinded [53].

#### 3.2.2. Risk of MACE Randomized Clinical Trials

**Figure 8:**
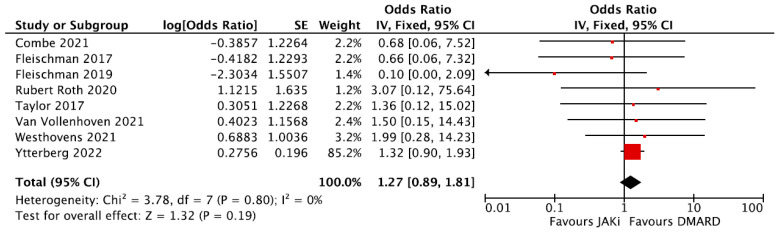
Forest plot for risk of MACE from clinical trials: DMARDs vs. JAKi. DMARDs = Disease-Modifying Anti-Rheumatic Drugs; JAKi = Janus Kinase inhibitors [46,47,48,49,50,51,52,53].

All eight RCTs provided data enabling the calculation of the odds ratio (OR) for MACE between patients receiving DMARDs and those receiving JAKi. The patient demographics and individual study results are summarized in Supplementary Tables S5 and S6. Fleischman et al. had the lower sample out of all the RCTs with 361 participants [48]. Ytterberg et al. had the higher sample with a total of 4362 patients: 2911 in the tofacitinib arm (across two cohorts taking 5 mg and 10 mg, respectively) and 1451 in the TNFi arm. The mean age was 61.0 years of age in all groups [53]. In all other seven RCTs, the mean age ranged from 51.9 to 55.8 between different treatment arms. The proportion of females included in different treatment groups was again high and ranged from 70.0% to 83.1%. The participant age and the percentage of female participants were similar across the different arms of each trial. Four RCTs compared JAKi to TNFi [46,48,50,53] three compared JAKi to MTX [47,51,52], and one compared JAKi to abatacept [49]. Overall, the incidence of MACE was no different in the intervention and comparator arms across all studies. A meta-analysis showed no evidence of heterogeneity and no statistically significant difference in the OR of MACE between the two groups, OR = 1.27, 95% CI = 0.89–1.81 (Figure 8).

#### 3.2.3. Risk of VTE Randomized Clinical Trials

**Figure 9:**
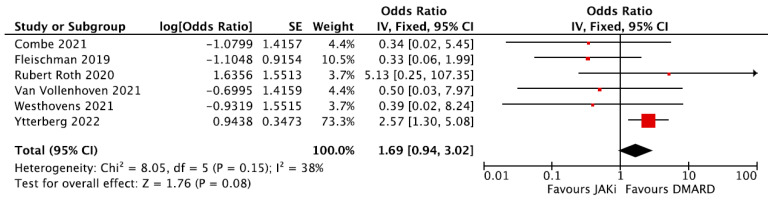
Forest plot of risk of VTE from RCTs: DMARDs vs. JAKi. DMARDs = Disease-Modifying Anti-Rheumatic Drugs; JAKi = Janus Kinase inhibitors; RCTs = Randomized Clinical Trials [46,48,49,51,53].

Six RCTs provided data to calculate the OR of VTE in a group of patients that used DMARDs and a group of patients that used JAKi [46,48,49,51,52,53]. Meta-analysis of these studies did not show a statistically significant difference in the odds of developing VTE in the JAKi group (pooled OR = 1.69, 95% CI = 0.94–3.02, Figure 9). It should be noted that out of the six clinical studies, four showed an OR < 1 and two an OR > 1, with the results mainly driven by the Ytterberg et al. study. In a sensitivity analysis excluding the Ytterberg et al. study, the results were convincingly not significant, with a pooled OR = 0.53, 95% CI = 0.17–1.6 and no evidence of heterogeneity, I^2^ = 0 (Supplementary Figure S3). It is also important to highlight that the Ytterberg et al. study used tofacitinib doses of 5 mg and 10 mg as two separate groups when comparing them against TNFi. However, in rheumatology practice, the dose is usually limited to 5 mg. Therefore, if we consider only the group which received 5 mg, the overall effect of JAKi on VTE remains non-significant (OR = 1.16, 95% CI = 0.61–2.22) (Supplementary Figure S4).

## 4. Discussion

Rheumatoid arthritis carries an increased cardiovascular risk which is driven by chronic inflammation and is exacerbated by the co-existence of other risk factors, such as diabetes, hypertension, and hyperlipidemia [54]. In addition, there is an increased VTE incidence in patients with RA, as inflammation suppresses endogenous anticoagulant agents and fibrinolysis, and upregulates procoagulants [55]. JAKi have been shown to increase VTE risk in RA patients; while the exact mechanism for this is poorly understood, a recent study suggested that JAKi augments immunothrombosis [56]. Whilst acknowledging the risks associated with JAKi, it needs to be emphasized that RA can substantially impair patients’ quality of life, with pain frequently limiting the ability to engage in routine and meaningful activities [57]. To this end, it is essential to use all available therapeutic strategies, including JAKi, to manage this disease effectively. JAKi can offer additional benefits in those patients with difficult-to-treat RA. This improved management of RA can in turn help with improved physical and mental status. Several studies have demonstrated a superior therapeutic response with JAKi compared with TNFi. In the study by Fleischmann et al. for example, a greater proportion of patients receiving upadacitinib achieved low disease activity or remission than those treated with adalimumab [48]. In another trial, baricitinib achieved a better ACR20 response compared to adalimumab [50]. In addition, a meta-analysis has shown that tofacitinib limited the radiographic progression of RA by up to 5 years [58]. In other studies, JAKi showed comparable effects to those of TNFi [23,26], and there have been no studies in which JAKi had an inferior treatment effect compared to other bDMARDs. Notwithstanding the potential benefit of JAKi, public bodies including the UK NICE have raised concerns regarding JAKi use in patients over 65 years of age, patients with cardiovascular risk factors, and smokers [6]. To our knowledge, this is the first meta-analysis that focuses on identifying the risks associated with JAKi, specifically in patients aged over 65 years old.

Our findings reassuringly demonstrate that the overall risk of MACE does not differ significantly between TNFi and bDMARDs when multivariable regression models are considered, indicating that whilst this can be a high-risk group of patients with multiple cardiac risk factors, JAKi use itself does not portend incremental risks when other risk factors are adjusted for. In the general RA population, our meta-analyses did not demonstrate a statistically significant increase in MACE in the JAKi group compared with DMARDs in either observational studies or RCTs. In contrast, we observed an increased VTE risk associated with JAKi relative to DMARDs in the observational studies, but not in the RCTs. This could potentially be because the observational studies tend to also include patients who are frailer and at higher risk, and who would not have been eligible or less likely to participate in RCTs. Therefore, it should be considered that JAKi may increase the risk of thromboembolism in routine clinical practice. However, any potential thromboembolic risk associated with JAKi should be weighed against their substantial therapeutic efficacy in the treatment of moderate to severe RA. Notably, other medications used commonly in RA and non-RA patients also carry recognized thrombotic risks, yet these remain widely used in clinical practice for their respective indications as the benefits outweigh the risks. To this end, we believe that JAKi should still be advocated for suitable RA patients, provided that a personalized medicine approach is adopted, and the increased risk is communicated with the patients in a shared-decision fashion. Moreover, other potential risk factors can be managed at the point of JAKi consideration in order to reduce the overall absolute risk for these patients. This requires consideration of patient characteristics, relevant biomarkers, disease phenotypes, and comorbidities [59], and can involve both pharmacotherapeutic and non-pharmacotherapeutic approaches [60]. In addition, it would also be appropriate to educate our patients on JAKi about these risks as well as early signs of VTE, so they can seek help earlier if any initial symptoms or signs of VTE are noticed.

### Implications for Personalized Cardiovascular Risk Stratifications in RA Patients Treated with JAKi

At a practical level, a personalized approach to the management of RA is becoming increasingly important as our understanding of disease heterogeneity continues to evolve. Patients differ substantially in their clinical presentation, comorbidity profiles, and treatment response, highlighting the need for therapeutic strategies tailored to individual risk, benefit, and preference. Importantly, patients with RA already have an inherently elevated risk of CVD, and those being considered for JAKi are often individuals with more severe disease, and therefore have a higher burden of cardiovascular comorbidity. This clinical context provides an important opportunity to perform a structured cardiovascular risk assessment considering age, prior VTE, smoking status, frailty, and a dedicated cardiovascular risk assessment. There are multiple cardiovascular assessment tools which can be used in patients with RA that can help identify higher risk patients who could benefit from additional pharmacotherapeutic and not pharmacotherapeutic measures. Such tools include PREVENT-CVD, PREVENT-ASCVD, SCORE2, and QRISK3, though a recent article suggested that QRISK3 performs less well for RA patients [61,62]. These can help implement comprehensive prevention strategies that combine lifestyle modifications, including nutrition, physical activity, and smoking cessation, with evidence-based pharmacotherapy, including aggressive lipid management in accordance with international guidelines such as from the European Society of Cardiology [63]. Within this personalized framework, JAKi remain a valuable therapeutic option, particularly for patients who have demonstrated inadequate response to conventional or biologic DMARDs, provided that the individual risk profile is carefully evaluated [64].

## 5. Limitations

In our meta-analysis, we included both observational studies, which may be susceptible to bias, and randomized clinical trials. For observational data, multivariable or propensity-matched regression models were used in preference to try to identify the independent effect of JAKi, and these were available for all the studies included in the meta-analysis.

Whilst a comparison with other pharmacotherapeutic agents used in managing RA, such as anti–IL-6 or anti-CD20 bDMARDs, would be clinically relevant, there was insufficient data available to permit the inclusion of a comparative analysis between JAKi and anti–IL-6 or anti-CD20 biologic DMARDs in this meta-analysis.

## 6. Conclusions

In this comprehensive systematic review and meta-analysis, we found no statistically significant difference in the incidence of MACE between patients treated with JAKi and those receiving DMARDs, and this remained unchanged in analyses limited to individuals older than 65 years—an area specifically investigated for the first time. However, JAKi use was associated with a significantly increased risk of VTE in observational studies, but not in the RCTs. JAKi are thus safe for MACE in appropriately selected patients, but require an individualized thrombotic risk mitigation. Our work emphasizes the need for a personalized management approach in patients eligible for JAKi therapy, a cohort characterized by high cardiovascular risk. This risk should be considered by clinicians when prescribing disease-modifying therapies, offering personalized medicine by considering factors such as disease stage, prior treatment response, patient preference, and the integration of both pharmacologic and nonpharmacologic strategies to reduce cardiovascular risks.

## Data Availability

The original contributions presented in this study are included in the article/Supplementary Material. Further inquiries can be directed to the corresponding author.

## Supplementary Materials

The following supporting information can be downloaded at: https://www.mdpi.com/article/10.3390/jpm16020113/s1, Supplementary Table S1 showing the full search strategy undertaken. Supplementary Table S2 PRISMA checklist. Supplementary Table S3: Demographics of participants in observational studies. Supplementary Table S4: Cumulative results presented in observational trials. Supplementary Table S5: Demographics as presented in RCT. Supplementary Table S6: Results as presented in RCT. Supplementary Figure S1: Risk of bias summary observational studies indicating overall low risk of bias in the majority of the studies. Supplementary Figure S2: Risk of bias summary for Randomized Clinical Trials, indicating low risk of bias. Supplementary Figure S3. Risk of VTE RCT. bDMARD vs JAKI sensitivity analysis without Ytterberg et al. Supplementary Figure S4. Risk of VTE RCT. bDMARD vs JAKI sensitivity analysis including only the Tofacitinib 5 mg group from Ytterberg et al.

## Abbreviations

The following abbreviations are used in this manuscript:

Abbreviation	Full Term
ABA	Abatacept
ADA	Adalimumab
BARI	Baricitinib
BDMARDs	Biological DMARDs
CSDMARDs	Conventional DMARDs
DMARDs	Disease-Modifying Drugs
DVT	Deep Vein Thrombosis
ETA	Etanercept
FIL	Filgotinib
HR	Hazard Ratio
IR	Incidence Rate
IRR	Incidence Rate Ratio
JAKi	Janus Kinase Inhibitors
MACE	Major Adverse Cardiovascular Event
MTX	Methotrexate
OR	Odds Ratio
PE	Pulmonary Embolism
RCT	Randomized Controlled Trial
ROR	Reported Odds Ratio
TNFi	Tumor Necrosis Factor Inhibitors
TOF	Tofacitinib
UPA	Upadacitinib
VTE	Venous Thromboembolism
