# Supplementary material for: Cardiovascular and Thromboembolic Risk of Janus Kinase Inhibitors Compared to Other Disease-Modifying Drugs in Patients with Rheumatoid Arthritis: A Systematic Review and Meta-Analysis
Source: J Pers Med. 2026 Feb 13;16(2):113. doi: 10.3390/jpm16020113 (PMC12941424; doi:10.3390/jpm16020113)
Supplement: Supplementary file 1 [file jpm-16-00113-s001.zip › jpm-4084137-supplementary 2.pdf]

Cardiovascular and thromboembolic risk of Janus Kinase inhibitors compared to other disease modifying drugs in patients with rheumatoid arthritis: A systematic review and meta-analysis

Diomidis C Ioannidis, Efthymia Maria Kapasouri, Vassilios S Vassiliou, Eleana Ntatsaki

Supplementary material

TABLES

Full search strategy

PUBMED	Arthritis, Rheumatoid[Mesh] OR rheumatoid arthritis OR RA) AND (Janus Kinase Inhibitors [Mesh] OR JAK inhibitors OR tofacitinib OR baricitinib OR upadacitinib OR filgotinib OR peficitinib) AND (cardiovascular [Mesh] OR Heart [Mesh] OR Cardiac [Mesh]).
EMBASE	(Arthritis, Rheumatoid or Rheumatoid Arthritis or RA) and Janus Kinase Inhibitors) or JAK inhibitors or baricitinib or filgotinib or

	upadacitinib or peficitinib) and cardiac) or heart or cardiovascular).
COCHRANE	(Rheumatoid arthritis OR RA) AND (Janus Kinase Inhibitors OR JAK inhibitors OR tofacitinib OR baricitinib OR upadacitinib OR filgotinib OR peficitinib) AND (cardiovascular OR Heart OR Cardiac)<br><br>Word variations have been searched

Supplementary Table S1 showing the full search strategy undertaken.

Section and Topic	Item #	Checklist item	Location where item is reported
**TITLE**			
Title	1	Identify the report as a systematic review.	Page 1
**ABSTRACT**			
Abstract	2	See the PRISMA 2020 for Abstracts checklist.	Page 1-2
**INTRODUCTION**			
Rationale	3	Describe the rationale for the review in the context of existing knowledge.	Page 2-5
Objectives	4	Provide an explicit statement of the objective(s) or question(s) the review addresses.	Page 3-4
**METHODS**			
Eligibility criteria	5	Specify the inclusion and exclusion criteria for the review and how studies were grouped for the syntheses.	Page 6
Information sources	6	Specify all databases, registers, websites, organisations, reference lists and other sources searched or consulted to identify studies. Specify the date when each source was last searched or consulted.	Page 5-6
Search strategy	7	Present the full search strategies for all databases, registers and websites, including any filters and limits used.	Supplement
Selection process	8	Specify the methods used to decide whether a study met the inclusion criteria of the review, including how many reviewers screened each record and each report retrieved, whether they worked independently, and if applicable, details of automation tools used in the process.	Page 7
Data collection process	9	Specify the methods used to collect data from reports, including how many reviewers collected data from each report, whether they worked independently, any processes for obtaining or confirming data from study investigators, and if applicable, details of automation tools used in the process.	Page 7
Data items	10a	List and define all outcomes for which data were sought. Specify whether all results that were compatible with each outcome domain in each study were sought (e.g. for all measures, time points, analyses), and if not, the methods used to decide which results to collect.	Page 7
	10b	List and define all other variables for which data were sought (e.g. participant and intervention characteristics, funding sources). Describe any assumptions made about any missing or unclear information.	Page 7, Supplement
Study risk of bias assessment	11	Specify the methods used to assess risk of bias in the included studies, including details of the tool(s) used, how many reviewers assessed each study and whether they worked independently, and if applicable, details of automation tools used in the process.	Page 7
Effect measures	12	Specify for each outcome the effect measure(s) (e.g. risk ratio, mean difference) used in the synthesis or presentation of results.	Page 7
Synthesis methods	13a	**Describe the processes used to decide which studies were eligible for each synthesis (e.g. tabulating the study intervention characteristics and comparing against the planned groups for each synthesis (item #5)).**	Page 9-15
	13b	Describe any methods required to prepare the data for presentation or synthesis, such as handling of missing summary statistics, or data conversions.	Page 9-15
	13c	Describe any methods used to tabulate or visually display results of individual studies and syntheses.	Page 9-15
	13d	Describe any methods used to synthesize results and provide a rationale for the choice(s). If meta-analysis was performed, describe the	Page 7

Section and Topic	Item #	Checklist item	Location where item is reported
		model(s), method(s) to identify the presence and extent of statistical heterogeneity, and software package(s) used.	
	13e	Describe any methods used to explore possible causes of heterogeneity among study results (e.g. subgroup analysis, meta-regression).	Page 7
	13f	Describe any sensitivity analyses conducted to assess robustness of the synthesized results.	Page 15
Reporting bias assessment	14	Describe any methods used to assess risk of bias due to missing results in a synthesis (arising from reporting biases).	n/a
Certainty assessment	15	Describe any methods used to assess certainty (or confidence) in the body of evidence for an outcome.	Page 16,17
**RESULTS**			
Study selection	16a	Describe the results of the search and selection process, from the number of records identified in the search to the number of studies included in the review, ideally using a flow diagram.	Page 8
	16b	Cite studies that might appear to meet the inclusion criteria, but which were excluded, and explain why they were excluded.	Page 8
Study characteristics	17	Cite each included study and present its characteristics.	Page 9, Page14, Supplement
Risk of bias in studies	18	Present assessments of risk of bias for each included study.	Supplement
Results of individual studies	19	For all outcomes, present, for each study: (a) summary statistics for each group (where appropriate) and (b) an effect estimate and its precision (e.g. confidence/credible interval), ideally using structured tables or plots.	Page 9-15, Supplement
Results of syntheses	20a	For each synthesis, briefly summarise the characteristics and risk of bias among contributing studies.	Page 9, Page 14
	20b	Present results of all statistical syntheses conducted. If meta-analysis was done, present for each the summary estimate and its precision (e.g. confidence/credible interval) and measures of statistical heterogeneity. If comparing groups, describe the direction of the effect.	Page 9, Page 15
	20c	Present results of all investigations of possible causes of heterogeneity among study results.	Supplement
	20d	Present results of all sensitivity analyses conducted to assess the robustness of the synthesized results.	Page 15
Reporting biases	21	Present assessments of risk of bias due to missing results (arising from reporting biases) for each synthesis assessed.	n/a
Certainty of evidence	22	Present assessments of certainty (or confidence) in the body of evidence for each outcome assessed.	Page 9-15
**DISCUSSION**			
Discussion	23a	Provide a general interpretation of the results in the context of other evidence.	Page 15-17
	23b	Discuss any limitations of the evidence included in the review.	Page 18
	23c	Discuss any limitations of the review processes used.	Page 7

Section and Topic	Item #	Checklist item	Location where item is reported
	23d	Discuss implications of the results for practice, policy, and future research.	Page 17-18
**OTHER INFORMATION**			
Registration and protocol	24a	Provide registration information for the review, including register name and registration number, or state that the review was not registered.	Page 5
	24b	Indicate where the review protocol can be accessed, or state that a protocol was not prepared.	Page
	24c	Describe and explain any amendments to information provided at registration or in the protocol.	n/a
Support	25	Describe sources of financial or non-financial support for the review, and the role of the funders or sponsors in the review.	Page 19
Competing interests	26	Declare any competing interests of review authors.	Page 19
Availability of data, code and other materials	27	Report which of the following are publicly available and where they can be found: template data collection forms; data extracted from included studies; data used for all analyses; analytic code; any other materials used in the review.	Data extracted from including studies, data collection used for analysis available on supplement

Supplementary Table S2 PRISMA checklist. *From:* Page MJ, McKenzie JE, Bossuyt PM, Boutron I, Hoffmann TC, Mulrow CD, et al. The PRISMA 2020 statement: an updated guideline for reporting systematic reviews. BMJ 2021;372:n71. doi: 10.1136/bmj.n71. This work is licensed under CC BY 4.0. To view a copy of this license, visit https://creativecommons.org/licenses/by/4.0/

STUDY	AGE	FEMALE %	SMOKING %	PAST MI/CVD %	PAST STROKE/ CEREBROVASCULAR %	HTN %	DIABETES %
**AYMON [21]**	**Mean**<br>**JAKi**<br>58.29 (12.22)<br>**TNFi**<br>57.12 (13.34)	**JAKi**<br>(78.3)<br>**TNFi**<br>(75.5)	**JAKi**<br>36%<br>**TNFi**<br>38%	**JAKi**<br>2%<br>**TNFi**<br>1.7%	**JAKi**<br>1.3%<br>**TNFi**<br>1,5 %	**JAKi**<br>30.4%<br>**TNFi**<br>27.4%	**JAKi**<br>9.1%<br>**TNFi**<br>8.8%
**BOWER [22]**	**MEDIAN IQR**<br>**JAKi**<br>60 (51–70)<br>**TOFA**<br>59 (50–68)<br>**BARI**<br>61 (51–71)<br>**UPA**<br>59 (50–69)<br>**TNFi**<br>58 (47–68)	**JAKi**<br>82<br>**BARI**<br>82<br>**TOF**<br>82<br>**UPA**<br>82<br>**TNFi**<br>77	**JAKi**<br>58<br>**BARI**<br>59<br>**TOF**<br>57<br>**UPA**<br>57<br>**TNFi**<br>57	-	**JAKi**<br>5<br>**BARI**<br>5<br>**TOF**<br>4<br>**UPA**<br>2<br>**TNFi**<br>3	-	-
**CHO SK 2023 [23]**	**Mean**<br>**TOF**<br>54.6 (43.8, 62.3)<br>**TNFi**<br>51.3 (39.9, 59.6)<br>p= 0.006	**TOF**<br>90.5<br>**TNFi**<br>84.6	-	-	**TOF**<br>0.5<br>TNFi<br>**0.2**	-	**TOF**<br>11<br>**TNFi**<br>6.2I
**CHO SK 2025 [25]**	**Mean**<br>**JAKi**<br>54.9+- 11.5<br>**bDMARDS**<br>52.5+-15<br>P 0.163	**JAKi**<br>85.7<br>**bDMARDS**<br>86.7	**JAKi**<br>18.4<br>**bDMARDS**<br>16<br>P=0.564	**JAKi**<br>0.7<br>**bDMARDS**<br>0.7<br>p=0.434	**JAKi**<br>0.7<br>**bDMARDS**<br>0.7<br>P=0.999	N/A	**JAKi**<br>5.3<br>**bDMARDS**<br>53<br>P=0.910
**CHO Y [24]**	**Mean (SD)**<br>**JAKi**<br>54.6 (13.2)<br>**TNFi**<br>54.6 (9.4)	**JAKi**<br>79.1<br>**TNFIi**<br>79.1	**JAKi**<br>14.8<br>**TNFi**<br>14.4	**JAKi**<br>3.1<br>**TNFi**<br>3.1	**JAKi**<br>3.1<br>**TNFi**<br>3,1	**JAKi**<br>24.9<br>**TNFi**<br>24.9	**JAKi**<br>13.1<br>**TNFi**<br>13.1
**DEAKIN [26]**	**Median (IQR)**<br>**TOF**<br>59 (51-68)<br>**ADA**<br>56(47-66)	**TOF**<br>73.6<br>**ADA**<br>68	-	-	-	-	-

**DESAI [27]**	**Mean (SD)**<br>**MarketScan**<br>**TOF**<br>53 (11.7)<br>**TNFi**<br>53 (12.3)<br>**Medicare**<br>**TOF**<br>71 (5.7)<br>**TNFi**<br>71 (5.5)<br>**Optum**<br>**TOF**<br>56 (12.7)<br>**TNFi**<br>56 (13.3)	**MarketScan**<br>**TOF**<br>82<br>**TNFi**<br>82.1<br>**Medicare**<br>**TOF**<br>86.6<br>**TNFi**<br>86.8<br>**Optum**<br>**TOF**<br>82.5<br>**TNFi**<br>82.8	-	**MarketScan**<br>**TOF**<br>5.2<br>**TNFi**<br>5.1<br>**Medicare**<br>**TOF**<br>15<br>**TNFi**<br>14.9<br>**Optum**<br>**TOF**<br>7<br>**TNFi**<br>6.8	**MarketScan**<br>**TOF**<br>0.7<br>**TNFi**<br>0.7<br>**Medicare**<br>**TOF**<br>2<br>**TNFi**<br>2.1<br>**Optum**<br>**TOF**<br>1.4<br>**TNFi**<br>1.	-	**MarketScan**<br>**TOF**<br>15.8<br>**TNFi**<br>15.7<br>**Medicare**<br>**TOF**<br>26.1<br>**TNFi**<br>26<br>**Optum**<br>**TOF**<br>20.9<br>**TNFi**<br>20.8
**FANG [28]**	**Mean (SD)**<br>**JAKi**<br>56.02<br>(12.11)<br>**TNFi**<br>55.81<br>(12.68)	**JAKi**<br>79.55<br>**TNFi**<br>78.09	-	-	-	**JAKi**<br>32.85<br>**TNFi**<br>33.8	**JAKi**<br>16.82<br>**TNFi**<br>16.94
**FRISELL [29]**	**Mean (SD)**<br>**TOFA**<br>59(13)<br>**BARI**<br>61(14)<br>**ETA**<br>58 (14)	**TOF**<br>82<br>**BARI**<br>82<br>**ETA**<br>77	-	**TOF**<br>1.6<br>**BARI**<br>2.6<br>**ETA**<br>1.7	**TOF**<br>0.9<br>**BARI**<br>1.3<br>**ETA**<br>1	-	**TOF**<br>8.9<br>**BARI**<br>8<br>**ETA**<br>7.2
**HIROSE [30]**	**Mean (SD)**<br>**JAKi**<br>68.5<br>**TNFi**<br>66.1<br>P- 0.1	**TOF**<br>82.3<br>**ABA**<br>85.4<br>P-0.22	-	-	-	-	-
**HOISNARD [31]**	**Mean (SD)**<br>**JAKi**<br>*59.3 (13.3)*<br>**TNFi**<br>*55.3 (13.4)*	**JAKi**<br>78.3<br>**TNFi**<br>71.2	**JAKi**<br>6.2<br>**TNFi**<br>6.1	**JAKi**<br>11.8<br>**TNFi**<br>8.5	-	**JAKi**<br>22.3<br>**TNFi**<br>20.2	**JAKi**<br>10.8<br>**TNFi**<br>8
**KHOSROW-KHAVAR [32]**	**Means (SD)**<br>**Optum**<br>**TOF**<br>56.8 (12.5)<br>**TNFi**<br>57.1 (13.2)<br>**Market Scan**<br>**TOF**<br>54.7 (11.5)<br>**TNFi**<br>55.0 (12.0)<br>**Medicare**<br>**TOF**<br>72.1 (5.6)<br>**TNFi**<br>72.2 (5.6)	**Optum**<br>**TOF**<br>80.0<br>**TNFi**<br>81.2<br>**Market Scan**<br>**TOF**<br>81.8<br>**TNFi**<br>81.8<br>**Medicare**<br>**TOF**<br>82.9<br>**TNFi**<br>83.3	**Optum**<br>**TOF**<br>19.9<br>**TNFi**<br>20<br>**Market Scan**<br>**TOF**<br>8.8<br>**TNFi**<br>8.9<br>**Medicare**<br>**TOF**<br>25.7<br>**TNFi**<br>25.6	**Optum**<br>**TOF**<br>10.1<br>**TNFi**<br>10.4<br>**Market Scan**<br>**TOF**<br>8.0<br>**TNFi**<br>8.3<br>**Medicare**<br>**TOF**<br>23.9<br>**TNFi**<br>23.7	**Optum**<br>**TOF**<br>2.4<br>**TNFi**<br>2.5<br>**Market Scan**<br>**TOF**<br>2.1<br>**TNFi**<br>2.2<br>**Medicare**<br>**TOF**<br>3.5<br>**TNFi**<br>3.5	**Optum**<br>**TOF**<br>52.3<br>**TNFi**<br>53<br>**Market Scan**<br>**TOF**<br>44.5<br>**TNFi**<br>45<br>**Medicare**<br>**TOF**<br>82.2<br>**TNFi**<br>82.1	**Optum**<br>**TOF**<br>21.4<br>**TNFi**<br>21.7<br>**Market Scan**<br>**TOF**<br>15.8<br>**TNFi**<br>15.9<br>**Medicare**<br>**TOF**<br>30.7<br>**TNFi**<br>30.5
**KREMER [33]**	**Mean (SD)**	**Unmatched population**	**Unmatched population**	**Unmatched population**	**Unmatched population**	**Unmatched population**	**Unmatched population**

	**Unmatched population**<br>**TOF**<br>59.6(12.1)<br>**BDMARD**<br>58.5(13)<br>**Trimmed population**<br>**TOF**<br>59.6 (12)<br>**bDMARD**<br>58.5 (12.9)	**TOF**<br>80.5<br>**bDMARD**<br>80.4<br>**PS Trimmed population**<br>**TOF**<br>80.7<br>**BDMARD**<br>80.1	**TOF**<br>19.6<br>**bDMARD**<br>18.1<br>**PS Trimmed population**<br>**TOF**<br>19.7<br>**bDMARD**<br>18.4	**TOF**<br>7.7<br>**bDMARD**<br>5.7<br>PS **Trimmed population**<br>**TOF**<br>7.8<br>**bDMARD**<br>5.9	**TOF**<br>3.4<br>**nbDMARD**<br>2.9<br>**PS Trimmed population**<br>**TOF**<br>3.5<br>**bDMARD**<br>3	**TOF**<br>35.5<br>**bDMARD**<br>32.3<br>**PS Trimmed population**<br>**TOF**<br>35.3<br>**bDMARD**<br>32.4	**TOF**<br>11.6<br>**bDMARD**<br>10.3<br>**PS trimmed population**<br>**TOF**<br>11<br>**bDMARD**<br>10.4
**MEISSNER [34]**	**Mean (SD)**<br>**JAKi**<br>60.2 (11.8)<br>**TNFi**<br>57.7(13.3)<br>**bDMARD**<br>60.4(11.9)<br>**csDMARD**<br>61.3 (12.3)	**JAKi**<br>74.4<br>**TNFi**<br>74<br>**bDMARD**<br>73.3<br>**csDMARD**<br>74.2	**JAKi**<br>25.9<br>**TNFi**<br>26.1<br>**bDMARD**<br>24.9<br>**csDMARD**<br>25.3	**JAKi**<br>8.2<br>**TNFi**<br>7<br>**bDMARD**<br>9.8<br>**csDMARD**<br>9.8	**JAKi**<br>2.4<br>**TNFi**<br>2.2<br>**bDMARD**<br>2.9<br>**csDMARD**<br>2.9	**JAKi**<br>46.7<br>**TNFi**<br>41.5<br>**bDMARD**<br>47.1<br>**csDMARD**<br>49.4	**JAKi**<br>13.9<br>**TNFi**<br>11.6<br>**bDMARD**<br>13.9<br>**csDMARD**<br>13.9
**MIN [35]**	**Mean (SD)**<br>**Set 1**<br>**JAKi**<br>52.3(12.8)<br>**TNFi**<br>50.16 (14.44)<br>**Set 2**<br>**JAKi**<br>51.5 (12.25)<br>**TNFi**<br>50.16 (14.44)	-	**Set 1**<br>**JAKi**<br>75.5<br>**TNFi**<br>72.3<br>**Set 2**<br>**JAKi**<br>83.2<br>**TNFi**<br>81.9	-	-	**Set 1**<br>**JAKi**<br>24<br>**TNFi**<br>24.4<br>**Set 2**<br>**JAKi**<br>27.1<br>**TNFi**<br>27.6	**Set 1**<br>**JAKi**<br>17.2<br>**TNFi**<br>15.7<br>**Set 2**<br>**JAKi**<br>15.9<br>**TNFi**<br>14.4
**MOK [36]**	**Mean (SD)**<br>**JAKi**<br>57.9 (11.4)<br>**TNFi**<br>52.6 (12.6)<br>P<0.001	**JAKi**<br>81.9<br>**TNFi**<br>84.3<br>P=0.18	**JAKi**<br>16.7<br>**TNFi**<br>15.1<br>P=0.36	**JAKi**<br>5.1<br>**TNFi**<br>2.3<br>P=0.001	**JAKi**<br>2.9<br>**TNFi**<br>2<br>P=0.22	**JAKi**<br>33<br>**TNFi**<br>23<br>P<0.001	**JAKi**<br>9.6<br>**TNFi**<br>5.8<br>P=0.002
**MOLANDER [37]**	**Median (IQR)**<br>**JAKi**<br>60 (51–70)<br>**TNFi**<br>57 (47–67)<br>**ABA**<br>60 (52–70)<br>–68)	**JAKi**<br>82<br>**TNFi**<br>77<br>**ABA**<br>80	**JAKi**<br>59<br>**TNFi**<br>58<br>**ABA**<br>62	**JAKi**<br>2<br>**TNFi**<br>2<br>**ABA**<br>3	**JAKi**<br>2<br>**TNFi**<br>2<br>**ABA**<br>3	-	**JAKi**<br>10<br>**TNFi**<br>9<br>**ABA**<br>10
**POPA [38]**	**Median (IQR) for total population**<br>62 (52-70)	**Total population**<br>72	-	-	-	**Total population**<br>35	**Total population**<br>11
**SAKAI [39]**	**Mean (SD)**<br>**JAKi**<br>65.3(13.6)<br>**BDMARDS**<br>63.4 (15)<br>**MTX**<br>65.2(13.7)	-	-	-	-	**JAKi**<br>20.8<br>**bDMARDS**<br>19<br>**MTX**<br>16.3	**JAKi**<br>7.6<br>**bDMARDS**<br>8.4<br>**MTX**<br>7.1
**SILVAGNI [40]**	**Median (IQR)**<br>**JAKi**<br>59 (51,68)<br>**TNFi**<br>57 (47,66)	**JAKi**<br>79.1<br>**TNFi**<br>80	-	**JAKi**<br>0.5<br>**TNFi**<br>0.5	**JAKi**<br>0.2<br>**TNFi**<br>0.4	**JAKi**<br>29.9<br>**TNFi**<br>25.9	**JAKi**<br>7.3<br>**TNFi**<br>6.7
**SONG YJ [42]**	**Mean (SD)**<br>**JAKi**<br>54.6<br>**TNFi**	**JAKi**<br>83.1<br>**TNFi**<br>80.2	-	-	-	-	-

	54.2						
**SONG YK [41]**	**Mean (SD)**<br>**Before PS Matching**<br>**JAKi**<br>48.1 (12.7)<br>**bDMARD**<br>48.5 (12.6)<br>**After PS matching**<br>**JAKi**<br>48.1 (12.7)<br>**bDMARD**<br>48.5 (12.6)	**Before PS Matching**<br>**JAKi**<br>70.6<br>**bDMARD**<br>73.1<br>**After PS matching**<br>**JAKi**<br>70.6<br>**bDMARD**<br>70.7	-	-	-	**Before PS Matching**<br>**JAKi**<br>57.3<br>**bDMARD**<br>58.7<br>**After PS matching**<br>**JAKi**<br>57.3<br>**bDMARD**<br>56.7	**Before PS Matching**<br>**JAKi**<br>22.3<br>**bDMARD**<br>24<br>**After PS matching**<br>**JAKi**<br>22.3<br>**bDMARD**<br>21.7
**TANAKA [43]**	**Mean (SD)**<br>**TOF**<br>61.3 (13.6)<br>**TNFI**<br>60.4 (15.0)<br>**MTX**<br>59.6 (14.3)<br>**NON TNFI bDMARD**<br>65.8 (12.4)	**TOF**<br>81.9<br>**TNFi**<br>80.6<br>**MTX**<br>74.6<br>**NON TNFi bDMARD**<br>75.5	**TOF**<br>9.2<br>**TNFi**<br>10.9<br>**MTX**<br>23.7<br>**NON TNFIibDMARD**<br>11	**TOF**<br>10.7<br>**TNFi**<br>8.8<br>**MTX**<br>8.7<br>**NON TNFi bDMARD**<br>13.2	-	**TOF**<br>27.8<br>**TNFi**<br>28.2<br>**MTX**<br>31.5<br>**NON TNFi bDMARD**<br>39.3	**TOF**<br>9.3<br>**TNFi**<br>7.5<br>**MTX**<br>9.1<br>**NON TNFi bDMARD**<br>13.5
**TONG [44]**	**Mean (SD)**<br>**JAKI**<br>53.8 (13.6)<br>**TNFi**<br>51.5 (15.6)	**JAKi**<br>78<br>**TNFi**<br>74.7	-	**JAKi**<br>9.7<br>**TNFi**<br>8.7	-	**JAKi**<br>30.9<br>**TNFi**<br>31.7	**JAKi**<br>19<br>**TNFi**<br>19.5
**UCHIDA [45]**	**Median (IQR)**<br>**TOF**<br>67 (58–73)<br>**BARI**<br>68 (58–75)<br>**TNFi**<br>51 (37–61)	**TOF**<br>80<br>**BAR**<br>82<br>**TNFi**<br>81	-	-	-	-	**TOFA**<br>19.8<br>**BARI**<br>24<br>**TNFi**<br>15.8

Supplementary Table S3: Demographics of participants in observational studies. ABA= Abatecept, BARI=Baricitinib, BDMARDs= biological DMARDS, CSDMARD= Conventional DMARD, ETA= Etarnecept JAKi= JAK Inhibitor , MTX= Methotrexate TNFi= TNF inhibitors, UPA= Upadacitinib.

STUDY	TYPE	COMPARISON	PARTICIPANT	MAIN RESULTS
**AYMON [21]**	Cross sectional	TNFI(Ref)Vs JAKi	**TNFI** 35,373<br>**JAKi**16,417	**Adjusted IRR (95% CI)**<br>**Combined data analysis**<br><br>**Overall population**<br>**Total MACE**<br>0.89 (0.63- 1.25)<br>**Stroke**<br>0.78 (0.42–1.46)<br>**MI**<br>0.91 (0.60–1.39)<br>**TIA**<br>1.36 (0.51–3.61)<br><br>**Adjusted IRR (95% CI)**<br>**Combined data analysis**<br>**MACE Age>50 with at least one cardiovascular risk factor**<br><br>1.1(0.55-2.22)<br><br>**Adjusted IRR (95% CI)**<br>**Combined data analysis**<br>**MACE History of cardiovascular disease**<br>1.16 (0.67-2.00)<br><br>**Adjusted HR MACE 95% CI**<br>0.93 (0.57 to 1.51)
**BOWER [22]**	Prospective cohort	TNFI (Ref) Vs JAKi	**TNFI 11,307**<br>**JAKiI** 3037<br>**TOF** 470<br>**BARI** 1973<br>**UPA** 587	**HR (95%CI) adjusted for confounders**<br><br>**MACE**<br>**TNFI (Ref) vs JAKi**<br>0.71 (0.51 to 0.99)<br>TNFi (Ref) vs TOFA<br>0.78 (0.39, 1.55)<br>**TNFI (Ref) vs BARI**<br>0.70 (0.49 to 1.01)<br><br>**ACUTE MI**<br><br>**TNFI (Ref) vs JAKi**<br>0.58 (0.36 to 0.94)<br>TNFi (Ref) vs TOFA<br>0.88 (0.35, 2.21)<br>**TNFI (Ref) vs BARI**<br>0.49 (0.28 to 0.88)<br><br>**STROKE**<br><br>**TNFI (Ref) vs JAKi**<br>0.67 (0.37 to 1.21)<br><br>TNFi(Ref) vs TOF<br>-<br>**TNFI (Ref) vs BARI**<br>0.83 (0.46 to 1.50)<br><br>0
**CHO SK 2025[25]**	Prospective Cohort	TNFI VS TOFA	**TNFi** 455<br>**TOF** 200	**Documented Cardiac disorders**<br>**TNFi**<br>N= 6<br>**TOF**<br>N= 4

**CHO SK 2023[23]**	Prospective Cohort	BDMARDS VS JAKi	**bDMARDs** 150<br>**TNFI** 87<br>**JAKi**196	**Documented Cardiac Disorders**<br>**bDMARD**<br>N=0<br>**JAKi**<br>N=3
**CHO Y[24]**	Retrospective Cohort	TNFi(Ref)VS JAKi	**bDMARDs** 150 (**TNFI** 87)<br>**JAKi**196	**3PM MACE Overall population**<br><br>**PROSPENSITY MATCHED HR**<br><br>**TNFi (Ref) Vs JAKi**<br>0.92 (0.59-1.42)<br>TNFi(Ref) vs TOF<br>0.87 (0.47,1.41)<br>TNFI (Ref) vs BARI<br>1.00 (0.53,1.87)
**DEAKIN [26]**	Retrospective Cohort<br>Trial Target Emulated	ADA VS TOF	**ADA** 569<br>**TOF** 273	**MACE**<br>**ADA**<br>N=0<br>**TOF**<br>N=1<br><br>**VTE**<br>**ADA**<br>N=1 (PE)<br>**TOF**<br>N=2 (1 PE, 1 DVT)
**DESSAI [27]**	Retrospective Cohort	TNFi(Ref) VS TOF	**TNFi** 80879<br>**TOF** 6774	**VTE Pooled HR (95% CI)**<br>1.13 (0.77- 1.65)<br><br>**PE HR (95% CI)**<br>1.02 (0.60- 1.73)<br><br>**DVT HR (95% CI)**<br>1.00 (0.79-1.26)
**FANG [28]**	Case Control	TNFi (Ref) VS JAKi	**TNFi** 822<br>**JAKi**2357	**Coronary heart disease HR 95% CI**<br><br>1.03 (0.45- 2.36),<br>P= 0.9463<br><br>**Stroke HR 95% CI**<br><br>0.75 (0.29- 1.94) P= 0.5519<br><br>**Overall venous Thromboembolism HR 95% CI**<br><br>0.65 (0.25- 1.70), p= 0.3810<br><br>**Deep vein thrombosis HR (95% CI)**<br><br>0.57 (0.20- 1.64), P =0.3010
**FRISELL [29]**	Case control	ETA (Ref) VS BARI<br>ETA (Ref) VS TOF	**ETA** 8748<br>**BARI** 1837<br>**TOF** 426	**MACE**<br>**HR (95%CI)**<br><br>**BARI VS ETA**<br>0.83 (0.49,1.42)<br><br>**TOF VS ETA**<br>0.78 (0.31,1.99)

**HIROSE [30]**	Prospective Cohort	ABA (Ref) VS TNFi	**ABA** 183<br>**TOF** 187	**0-52 weeks**<br>**Absolute numbers**<br>**MACE**<br>ABA N=1<br>TOF N=2<br>p- 0.49<br><br>**VTE**<br>ABA N=1<br>TOF N=0<br>P0.49
**HOISNARD [31]**	Retrospective Cohort	ADA (Ref) VS JAKi	**ADA** 7354<br>JAK**i** 8481	**MACE Adjusted HR (95% CI)**<br><br>**ADA VS JAKi. Total population**<br><br>1.0 (07, 1.5) , p =0.99<br><br>**ADA VS TOF Total population**<br><br>0.8 (0.4 , 1.4), p=0.44<br><br>**ADA VS BARI Total population**<br>1.1 (0.7, 1.8), p= 0.59<br><br><br>**JAKi vs ADA over 65 y.o with at least one CVD risk**<br>0.7 (0.4 , 1.3), p =0.26
**KHOSROW-KHAVAR [32]**	1)Real word evidence Cohort (RWE)<br>2) RCT-Duplicate cohort	TNFi (Ref) vs TOF	**RWE COHORT**<br>**TNFi** 89411<br>**TOF** 12852<br><br>**RCT-Duplicate**<br>**TNFi** 31573<br>**TOF** 3497	**MACE HR (95% CI)**<br><br>**RWE overall population**<br>1.01 (0.83, 1.23)<br><br>**RCT -duplicate overall population**<br>1.24 (0.90-1.69)<br><br>**RWE prior cardiac risk factors**<br>1.27(0.95-1.70)<br><br>**RWE >65 y.o**<br>1.05 (0.84-1.33)
**KREMER [33]**	Prospective cohort	bDMARD (Ref) vs TOF	**PS Unmatched**<br>**bDMARD** 8358<br>**TOF** 1999<br>**bDMARD** 7767<br>**TOF** 1866	**MACE unmatched population- IR per 100 PY (95% CI)**<br><br>**bDMARD**<br>0.88 (0.72-1.06)<br>**TOF**<br>0.70 (0.44-1.06)<br><br>**Stroke /TIA - IR per 100 PY (95% CI)**<br><br>**bDMARD**<br>0.47 (0.36-0.60)<br>**TOF**<br>0.32 (0.15-0.58)<br><br>**MACE PS trimmed population- IR per 100 PY (95% CI)**<br><br>**bDMARD**<br>0.91 (0.74-1.09)<br>**TOF**<br>0.64 (0.39-1.00)<br><br>**MACE PS trimmed population adjusted HR (95% CI)**

				0.61 (0.34-1.06)
**MEISNER [34]**	Retrospective cohort	bDMARD (Ref) VS JAKi<br>TNFI (ref) vs JAKi<br>csDMARD (Ref)	**TNFi**<br>3694<br>**Other bDMARD**<br>3150<br>**csDMARD**<br>4301<br>**JAKi**<br>3058	**MACE HR (95% CI)**<br><br>**TNFi v JAKi Overall population**<br><br>0.89 (0.52, 1.52)<br><br>**TNFI VS JAKi Age >65 y.o**<br>1.07(0.51-2.21)
**MIN [35]**	Retrospective cohort	TNFi (ref) vs JAKi	**SET 1- Patients never on bDMARD or JAKi**<br>**TNFi** 951<br>**JAKi** 645<br>**SET 2 Patient who previously used JAKi or bDMARD**<br>**TNFI** 9267<br>**JAKi** 2496	**MACE SET 1 HR (95% CI)- Overall population**<br>0.59 (0.35, 0.99) p=0.04<br><br>**MACE SET 1 HR (95% CI) >65 y.o**<br>0.66 (0.31, 1.41)<br><br>**MACE SET 2 HR (95% CI)- Overall population**<br>0.80 (0.67, 0.97)<br><br>**MACE SET 2 HR (95% CI) >65 y.o**<br>0.85 (0.64, 1.12)<br><br>**VTE SET 1 HR (95% CI)- Overall population**<br>0.33 (0.07, 1.54)<br><br>**VTE SET 2 (HR 95% CI)- Overall population**<br>1.34 (0.90, 1.99)
**MOK [36]**	Retrospective Cohort	TNFi (ref) vs JAKi	**TNFi** 1920<br>**JAKi** 551	**MACE Adjusted HR (95% CI)**<br>1.36(0.62-2.96), P=0.44<br><br>**MACE IRR (95% CI)**<br>1.49 (0.79, 2.84), p=0.22<br><br>**VTE IRR (95% CI)**<br>3.90 (0.20, 78.1), P=0.34
**MOLANDER [37]**	Prospective Cohort	TNFi (ref) vs JAKi<br>TNFi (ref) vs TOF<br>TNFi (ref) vs BARI	**TNFi** 19950<br>**JAKi**2354<br>**BARI** 1825<br>**TOF** 424	**VTE Adjusted HR (95% CI)**<br><br>**TNFi VS JAKi**<br>1.73 (1.24, 2.42)<br>**TNFi VS BARI**<br>1.79 (1.25, 2.55)<br>**TNFi VS TOF**<br>1.66 (0.77, 3.59)<br><br>**PE Adjusted HR (95% CI)**<br>**TNFi VS JAKi**<br>3.21 (2.11, 4.88)<br><br>**DVT Adjusted HR (95% CI)**<br>**TNFi VS JAKi**<br>0.83 (0.47-1.45)
**POPA [38]**	Retrospective Cohort	bDMARD(ref)vs JAKi	**bDMARD**<br>18882	**IRR (95% CI)**

			**JAKi** 2090	**TNFi vs JAKi**<br>1.01 (0.71, 1.43; 0.965)<br><br>**TNFi VS TOF**<br><br>1.02 (0.61, 1.72; 0.933)<br><br>**TNFi VS BARI**<br><br>1.05 (0.67, 1.64; 0.825)
**SAKAI [39]**	Retrospective cohort	TNFI (ref) VS JAKi<br>Non-	**bDMARD**<br>20694<br>**MTX**<br>37926<br>**JAKi**<br>4416	**Total cardiovascular events adjusted HR (95% CI)**<br><br>**TNFi**<br>**without MTX VS**<br>**JAKi without MTX**<br>1.66 (1.1, 2.49), P=0.013<br>**MTX vs JAKi without MTX**<br>1.19 (0.83, 1.71), p=0.34<br>**TNFi with MTX vs JAKI**<br>**with MTX**<br>1.72 (1.11, 2.66) P=0.015<br>**Non TNFi without MTX vs JAKi without MTX**<br>1.67 (1.13, 2.48) p= 0.010<br>**Non TNFi with MTX vs JAKi with MTX**<br>0.97 (0.63, 1.50) P=0.880
**SILVAGNI [40]**	Retrospective Cohort	TNFi(Ref) vs JAKi	**TNFi** 2343<br>**JAKi** 1443	**MACE ADJUSTED HR (95% CI)**<br>0.71 (0.77, 1.33) p= 0.282<br><br>**VTE ADJUSTED HR (95% CI)**<br>1.53 (0.65, 3.65) p= 0.0.332<br><br>**CVE ADJUSTED HR (95% CI)**<br>0.92 (0.64-1.32), p=0.661
**SONG YJ [42]**	Retrospective Cohort	TNFi (Ref) vs JAKi	**TNFi** 3307<br>**JAKi** 871	**VTEADJUSTED HR (95% CI)**<br>0.18 (0.01, 3.47)
**SONG YK [41]**	Retrospective Cohort	bDMARD (Ref) vs JAKi	**bDMARD**<br>3384<br>**JAKi** 846	**MACE ADJUSTED HR (95% CI) overall population**<br><br>**bDMARD vs JAKi**<br>1.28(0.53, 3.11)<br><br>**MACE ADJUSTED HR 95% CI >65 y.o**<br><br>**bDMARD vs JAKi**<br>1.33 (0.36, 9.31)
**TANAKA [43]**	Prospective Cohort	MTX VS TOF<br>NON TNFi VS TOF<br>TNFi VS TOFA	**MTX** 298<br>**NON TNFi** 758<br>**TNFi** 663<br>**TOF** 253	**MACE ADJUSTED IR (95% CI)**<br><br>**MACE**<br><br>**MTX**<br>0.34 (0.00, 0.83); **n**=2<br><br>**NON TNFi**<br>0.77 (0.35, 1.19); ***n*** = 14<br><br>**TNFi**<br>0.09 (0.00, 0.27); ***n*** = 1<br><br>**TOF**<br>0.48 (0, 1.20); ***n*** = 2<br><br>**VTE**<br><br>**MTX**<br>**n**=0

				**NON TNFi**<br>0.11 (0.00-0.22), **n=**2<br><br>**TNFi**<br>0.07 (0.00,0.2) **,n=**1<br><br>**TOFA**<br>0.55 (0.00,1.92), **n=**1
**TONG [44]**	Retrospective Cohort	TNFi(ref) vs JAKi	**TNFi** 5849<br><br>**JAKi** 1571	**CVE**<br>**adjusted risk ratio (95% CI)**<br><br>0.80 (0.48,1.35)
**UCHIDA [45]**	Retrospective Cohort	TNFi vs TOF<br>TNFi vs JAKI	**TNFi** 203<br>**TOF** 192<br>**BARI** 104	**MACE**<br>**Absolute numbers**<br>**TNFi** 0<br>**TOF** 2<br>**BARI** 0

Supplementary Table S4: Cumulative results presented in observational trials. IR – Incident rate, IRR (incidence rate ratio), ROR reporting odds ratio. ABA-> Abatecept, BARI-> Baricitinib, BDMARDs -> biological DMARDS, CSDMARD-> Conventional DMARD, ETA-> Etarnecept JAKI-> JAK Inhibitor , MTX-> Methotrexate TNFI-> TNF inhibitors, UPA-> Upadacitinib

STUDY	AGE	FEMALE %	SMOKING %	PAST MI/CVD %	PAST STROKE/ CEREBROVASCULAR %	HTN %	DIABETES %
**COMBE 2021 FINCH 1 [46]**	**Mean (SD)**<br>**FIL200**<br>52.2(12.8)<br>**FIL100**<br>53(12.6)<br>**ADA**<br>53(12.9)	**FIL200**<br>79.8<br>**FIL100**<br>83.1<br>**ADA**<br>81.8	-	-	-	-	-
**FLEISCHMANN 2017 RA- BEGIN [47]**	**Mean (SD)**<br>**BARI**<br>51(13)<br>**MTX**<br>51(13)	**BARI**<br>70<br>**MTX**<br>76	-	-	-	-	-
**FLEISCHMANN 2019 [48]**	**Mean (SD)**<br>**UPA**<br>54(12)<br>**ADA**<br>54(12)	-	-	-	-	-	-
**RUBBERT ROTH 2020 SELECT DECIDE [49]**	**Mean (SD)**<br>**UPA**<br>55.3 (11.4)<br>**ABA**<br>55.8<br>(11.9)	**UPA**<br>82.2<br>**ABA**<br>81.9	-	-	-	-	-
**TAYLOR 2017 RA-BEAM [50]**	**Means (SD)**<br>**BARI**<br>54(2)<br>**ADA**<br>53(12)	**BARI**<br>77<br>**ADA**<br>76	-	-	-	-	-
**VAN VOLLENHOVEN SELECT EARLY [51]**	**Mean (SD)**<br>**UP15**<br>51.9(12.6)<br>**UPA 30**<br>54.9(12.6)<br>**MTX**<br>53.3(12.9)	**UPA 15**<br>76<br>**UPA 30**<br>76.4<br>**MTX**<br>76.4	-	-	-	-	-
**WESTHOVENS FINCH 3 [52]**	**Mean (SD)**<br>**FIL200+MTX**<br>53 (13.8)<br>**FIL100+MTX**<br>54 (12.6)<br>**FIL200**<br>52 (13.9)<br>**MTX**<br>53 (13.7)	**FIL200+MTX**<br>78<br>**FIL100+MTX**<br>79<br>**FIL200**<br>75<br>**MTX**<br>77	**FIL200+MTX**<br>72<br>**FIL100+MTX**<br>71<br>**FIL200**<br>72<br>**MTX**<br>69	-	-	-	-
**YTTERBERG ORAL - SURVEILLANCE [53]**	Mean (SD)<br>**TOF 5 BD**<br>60.8 (6.8)<br>**TOF 10 BD**<br>61.4(7.1)<br>**TNFI**<br>61.3 (7.5)	**TOF 5 BD**<br>80.3<br>**TOF 10 BD**<br>77.2<br>TNFi<br>77	**TOFA 5 BD**<br>49.5<br>**TOFA 10 BD**<br>48.4<br>**TNFi**<br>46.8	**TOF 5 BD**<br>11.1<br>**TOF 10 BD**<br>11.8<br>**TNFi**<br>11.3	-	**TOF 5 BD**<br>65.6<br>**TOF 10 BD**<br>65.5<br>**TNFi**<br>66.8	**TOF 5 BD**<br>16.7<br>**TOF 10 BD**<br>17.9<br>**TNFi**<br>17.6

Supplementary Table S5: Demographics as presented in RCT
ABA= Abatacept, ADA= adalimumab, FIL100= Filgotinib 100mg once daily, FIL200= Filgotinib 200mg once daily, MTX= Methotrexate, TOF5= Tofacitinib 5 mg twice daily, TOF10= Tofacitinib 10 mg BD, UPA15= Upadacitinib 15 mg once daily, UPA30= Upadacitinib 30 mg once daily

STUDY	TYPE	COMPARISON	PARTICIPANTS	RESULTS
**COMBE 2021**<br>**FINCH 1 [46]**	Randomized control clinical trial	ADA VS FIL	**ADA**<br>325<br>**FIL100**<br>480<br>**FIL200**<br>475	**MACE**<br>**Absolute numbers at week 52**<br>**ADA** n=1<br>**FIL100** n=2<br>**Fil200** n=0<br><br>**VTE**<br>**Absolute numbers at week 52**<br>**ADA** n=1<br>**FIL100** n=1<br>**Fil200** n=0
**FLEISCHMANN 2017**<br>**RA- BEGIN [47]**	Double blind Randomized control clinical trial	MTX VS BARI	**MTX**<br>210<br>**BARI**<br>159	**MACE**<br>**Absolute numbers at week 52**<br>**MTX** n=2<br>**BARI** n=1
**FLEISCHMANN 2019 [48]**	Double blind Randomized control clinical trial	ADA VS UPA	**MTX+ ADA**<br>325<br>**MTX+ UPA**<br>650	**MACE Absolute numbers at week 26**<br>**ADA n=2**<br>**UPA n=0**<br><br>**VTE Absolute numbers at week 26**<br><br>**ADA n=3**<br>**UPA n=2**
**RUBBERT ROTH 2020**<br>**SELECT DECIDE [49]**	Double blind Randomized control clinical trial	ABA VS UPA	**ABA**<br>309<br>**UPA**<br>303	**MACE**<br>**Absolute numbers at week 24**<br>**ABA** n=0<br>**UPA** n=1<br><br>**VTE**<br>**Absolute numbers at week 24**<br><br>**ABA** n=0<br>**UPA** n=2
**TAYLOR 2017**<br>**RA BEAM [50]**	Double blind Randomized control clinical trial	ADA VS BARI	**ADA**<br>330<br>**BARI**<br>487	**MACE**<br>**Absolute numbers at week 52**<br>**ADA n=**1<br>**BARI** n=2
**VAN VOLLENHOVEN**<br>**SELECT EARLY [51]**	Randomised control clinical trial	MTX VS UPA	**MTX**<br>314<br>**UPA15**<br>314<br>**UPA30**<br>317	**MACE**<br>**Absolute numbers**<br><br>**MTX** n=1<br>**UPA15** n=1<br>**UPA30** n=2<br><br>**VTE**<br>**Absolute numbers**<br><br>**MTX**<br>n=1<br>**UPA15**<br>n=0<br>**UPA30**<br>n=1

**WESTHOVENS FINCH 3 [52]**	Double Blind Randomised Control Clinical Trial	MTX VS FIL	**MTX** 416<br>**FIL200** 210<br>**FIL100+MTX** 207<br>**FIL200+MTX** 416	**MACE**<br>**Absolute number at week 52**<br>**MTX** n=2<br>**FIL200** n=2<br>**FIL100+MTX** n=1<br>**FIL200+MTX** n=4<br><br>**VTE**<br>**Absolute number at week 52**<br><br>**MTX** n=2<br>**FIL200** n=0<br>**FIL100+MTX** n=0<br>**FIL200+MTX** n=0
**YTTERBERG ORAL - SURVEILLANCE [53]**	3b/4 randomised, open-label, non-inferiority,	TNFI (ref) VS TOF	**TNFI** 1451<br>**TOF5** 1455<br>**TOF10** 1456	**MACE at 60 days**<br><br>**HR (95% CI)**<br><br>**TOF5 VS TNFi**<br>1.24 (0.81-1.91)<br>**TOF10 VS TNFi**<br>1.43 (0.94-2.18)<br>**TOF COMBINED VS TNFi**<br>1.33 (0.91-1.94)<br><br>**Total events**<br>**TNFi** n= 37<br>**TOFA** combined n= 98<br><br>**VTE at 28 days**<br><br>**HR (95% CI)**<br><br>**TOFA5 VS TNFi**<br>1.66 (0.76–3.63)<br><br>**TOFA10 VS TNFi**<br>3.52 (1.74–7.12)<br><br>**TOFA combined vs TNFi**<br>2.56 (1.30, 5.05)<br><br>**Total events**<br>TNFi n= 10<br>TOFA Combined n= 51<br><br>**PE at 28 days HR**<br><br>**HR 95% CI**<br><br>**TOF5 VS TNFi**<br>2.93 (0.79–10.83)<br>**TOF10 VS TNFi**<br>8.26 (2.49–27.43)

	**TOF combined VS TNFi**<br>5.53 (1.70, 18.02)<br><br>**Total events**<br>**TNFi** n= 2<br>TOF combined n=3

Supplementary Table S6: Results as presented in RCT. ABA= Abatacept, ADA= adalimumab, FIL100= Filgotinib 100mg once daily, FIL200= Filgotinib 200mg once daily , MTX= Methotrexate, TOF5= Tofacitinib 5 mg twice daily, TOF10= Tofacitinib 10 mg BD, UPA15= Upadacitinib 15 mg once daily, UPA30= Upadacitinib 30 mg once daily

FIGURES

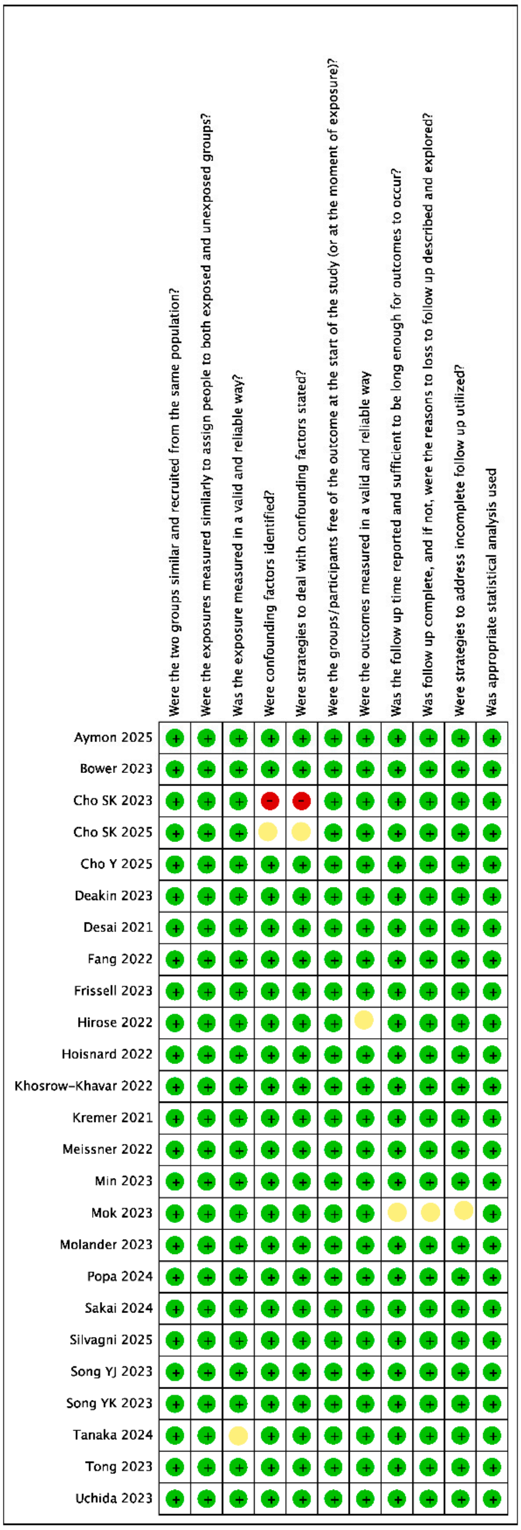

Supplementary Figure S1: Risk of bias summary observational studies indicating overall low risk of bias in the majority of the studies. [21-45]

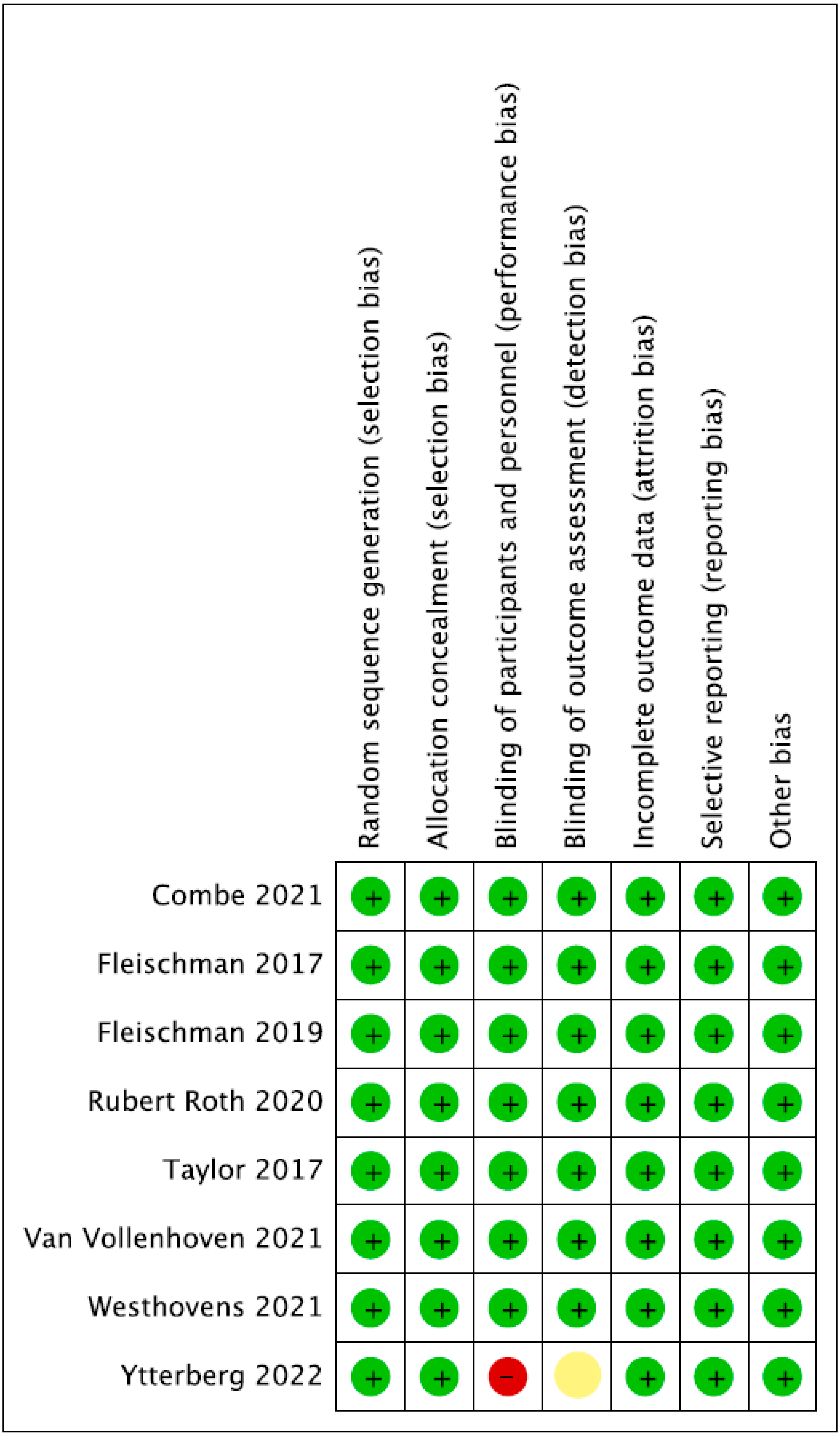

Supplementary Figure S2: Risk of bias summary for Randomized Clinical Trials, indicating low risk of bias. [46-53]

Study or Subgroup	log[Odds Ratio]	SE	Weight	Odds Ratio IV, Fixed, 95% CI
Combe 2021	−1.0799	1.4157	16.5%	0.34 [0.02, 5.45]
Fleischman 2019	−1.1048	0.9154	39.5%	0.33 [0.06, 1.99]
Rubert Roth 2020	1.6356	1.5513	13.7%	5.13 [0.25, 107.35]
Van Vollenhoven 2021	−0.6995	1.4159	16.5%	0.50 [0.03, 7.97]
Westhovens 2021	−0.9319	1.5515	13.7%	0.39 [0.02, 8.24]
**Total (95% CI)**			**100.0%**	**0.53 [0.17, 1.64]**

Heterogeneity: $Chi^2 = 2.54$, df = 4 (P = 0.64); $I^2 = 0\%$
Test for overall effect: Z = 1.10 (P = 0.27)

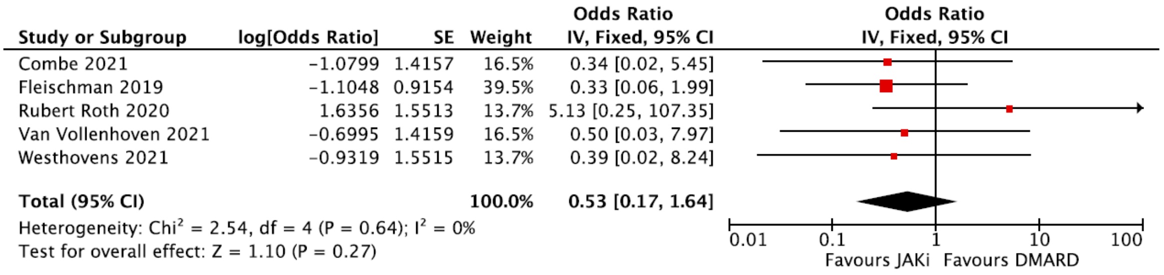

Supplementary Figure S3. Risk of VTE RCT. bDMARD vs JAKI sensitivity analysis without Ytterberg et al. [46, 48, 49 ,50, 51]

Study or Subgroup	log[Odds Ratio]	SE	Weight	Odds Ratio IV, Fixed, 95% CI
Combe 2021	−1.0799	1.4157	5.4%	0.34 [0.02, 5.45]
Fleischman 2019	−1.1048	0.9154	12.9%	0.33 [0.06, 1.99]
Rubert Roth 2020	1.6356	1.5513	4.5%	5.13 [0.25, 107.35]
Van Vollenhoven 2021	−0.6995	1.4159	5.4%	0.50 [0.03, 7.97]
Westhovens 2021	−0.9319	1.5515	4.5%	0.39 [0.02, 8.24]
Ytterberg 2022	0.5327	0.4003	67.4%	1.70 [0.78, 3.73]
**Total (95% CI)**			**100.0%**	**1.16 [0.61, 2.22]**

Heterogeneity: $Chi^2 = 5.31$, df = 5 (P = 0.38); $I^2 = 6\%$
Test for overall effect: Z = 0.46 (P = 0.64)

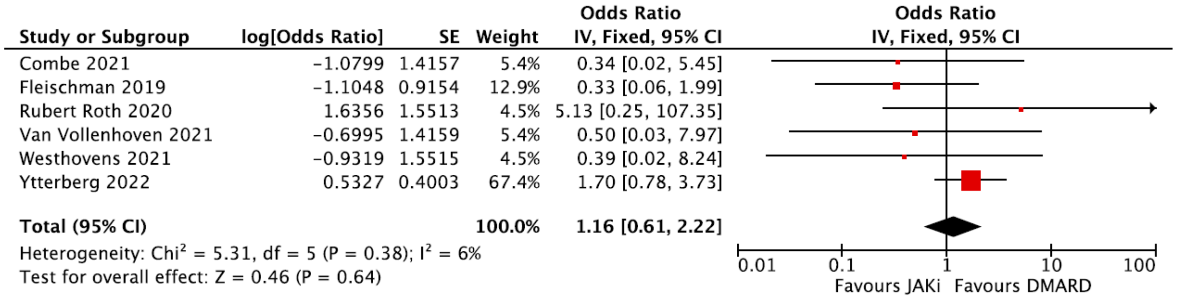

Supplementary Figure S4. Risk of VTE RCT. bDMARD vs JAKI sensitivity analysis including only the Tofacitinib 5 mg group from Ytterberg et al. [46, 48, 49 ,50, 51, 53]
